# Identification of Pre-symptomatic Gene Signatures That Predict Resilience to Cognitive Decline in the Genetically Diverse AD-BXD Model

**DOI:** 10.3389/fgene.2019.00035

**Published:** 2019-02-06

**Authors:** Sarah M. Neuner, Sarah E. Heuer, Ji-Gang Zhang, Vivek M. Philip, Catherine C. Kaczorowski

**Affiliations:** ^1^University of Tennessee Health Science Center, Memphis, TN, United States; ^2^The Jackson Laboratory, Bar Harbor, ME, United States; ^3^Tufts University Sackler School of Graduate Biomedical Sciences, Boston, MA, United States

**Keywords:** resilience, network analysis, Alzheimer’s, susceptibility, genetics, transcriptomics

## Abstract

Across the population, individuals exhibit a wide variation of susceptibility or resilience to developing Alzheimer’s disease (AD). Identifying specific factors that promote resilience would provide insight into disease mechanisms and nominate potential targets for therapeutic intervention. Here, we use transcriptome profiling to identify gene networks present in the pre-symptomatic AD mouse brain relating to neuroinflammation, brain vasculature, extracellular matrix organization, and synaptic signaling that predict cognitive performance at an advanced age. We highlight putative drivers of these observed relationships, including *Itgb2*, *Fcgr2b, Slc6a14*, and *Gper1*, which represent prime targets through which to promote resilience prior to overt symptom onset. In addition, we identify a genomic region on chromosome 2 containing variants that directly modulate resilience network expression. Overall, work here highlights new potential drivers of resilience to AD and contributes significantly to our understanding of early, potentially causal, disease mechanisms.

## Introduction

Alzheimer’s disease (AD) is the most common neurodegenerative disease, characterized by a combination of severe memory impairment and two classical neuropathologies, extracellular amyloid plaques and intracellular neurofibrillary tangles (Selkoe, 1991). According to the amyloid hypothesis, which has been extensively researched for decades, the formation and deposition of amyloid, particularly the toxic 1-42 amino acid species of beta-amyloid (Aβ1-42), is thought to be an initiating factor that leads to later neurodegeneration and cognitive impairment (Hardy and Selkoe, 2002; Selkoe and Hardy, 2016). However, many imaging and post-mortem studies of human brains have shown that substantial amounts of AD pathology, particularly plaque pathology, can be present in the brains of cognitively intact individuals (Morris et al., 1996; Negash et al., 2013). These individuals, who often meet the criteria for a pathological diagnosis of AD but remain asymptomatic, represent a clinically interesting subset of the population that exhibit a certain degree of resilience to what are typically highly deleterious neuropathologies.

Resiliency, defined as better cognitive functioning than predicted based on given pathological, genetic, or molecular characteristics, has been observed in both the general population (Hohman et al., 2016) and in families harboring high-risk genetic mutations that often confer early-onset or familial AD [FAD, (Ryman et al., 2014)]. While FAD is typically thought to be a severe form of the disease with an age of onset before 65, there is a wide range in the age at first symptom onset (Ryman et al., 2014). In both sporadic late-onset AD (LOAD) and FAD, disease onset is highly heritable (Gatz et al., 1997), indicating genetic factors likely play a large role in determining individual susceptibility or resilience. Identifying these specific genetic factors, particularly those that promote resilience, would provide key insight into disease mechanisms and nominate putative targets for therapeutic intervention, as strategies that delay disease onset even by a few years would provide much needed disease modifying therapies.

Despite the immense therapeutic potential presented by resilience factors, their identification in human populations remains elusive. Several studies have utilized FAD populations to identify modifiers of the age at first symptom onset (Lee et al., 2015), but these populations are typically not large enough to support genome-wide testing and identification of resilience factors. In addition, the identification of individuals with little to no family history of AD and who remain cognitively intact despite high pathology loads is almost impossible in the general population, as these individuals rarely enter the clinic. Even when resilient individuals enroll as part of an observational study, human-oriented research presents additional challenges. The human genome is incredibly complex, individuals are consistently exposed to a variety of uncontrollable environmental factors, and accessing critical disease-relevant tissue at early stages of disease is uncommon.

To overcome some of the barriers associated with studying AD in human populations, our lab and others have turned to the mouse as a model organism with which to study disease pathogenesis. FAD is often caused by inherited mutations in the genes encoding for amyloid precursor protein (*APP*) and presenilin 1 (*PSEN1*), and the cognitive and pathological symptoms observed strongly resemble those seen in late-onset AD (LOAD), the more prominent form of AD (Duara et al., 1993; Lippa et al., 1996; Day et al., 2016). As such, mouse models carrying human mutations in *APP* and/or *PSEN1* have emerged as a powerful way to study aspects of the human disease (Elder et al., 2010; Webster et al., 2014; Neuner et al., 2018). In addition, within the lab we can control environmental variables and access disease-relevant tissue at early disease time points, which is critical for understanding molecular mechanisms that drive AD-related cognitive decline. However, most AD mouse models utilize a single inbred strain (or single mixed background non-inbred strain), which precludes the identification of genetic factors underlying differential susceptibility or resilience to AD. To address this, our lab has developed the first genetically diverse transgenic AD mouse population (Neuner et al., 2018). This population, which we termed the AD-BXDs, combines two well-established resources, the 5XFAD transgenic model of AD (Oakley et al., 2006) and the BXD genetic reference panel (Peirce et al., 2004). The BXD genetic reference panel is a series of recombinant inbred mouse strains initially derived from a cross between the two common inbred strains C57BL/6J (B6) and DBA/2J (D2). As approximately 5 million polymorphisms segregate across these two strains (Wang et al., 2016), the BXD panel incorporates a substantial amount of genetic diversity into our studies but reduces complexity just enough to allow for well-powered genome-wide trait mapping with reasonable sample sizes. In addition, as each parental stain of this cross is fully inbred (B6.Cg-5XFAD, #34848-JAX, and each individual BXD strain), this approach allows for the rapid generation of genetically identical F1 AD-BXD mice, enabling repeated sampling across time and laboratories.

Here we take advantage of the inbred nature of the AD-BXD panel and identify transcriptional networks present at early stages of disease (6 months) that predict cognitive impairment later in disease (14 months). At 6 months of age, the AD-BXDs are cognitively unimpaired as a population relative to their non-transgenic littermates (Neuner et al., 2018) as measured by contextual fear conditioning, making the six-month time point ideal to profile networks present prior to overt symptom onset. Understanding the molecular mechanisms that occur early in disease may help to identify causal drivers of disease pathogenesis and therapeutic targets for interventions. As there is currently no cure of AD, work here is poised to contribute significantly to human health.

## Materials and Methods

### Bioethics

All mouse experiments occurred at University of Tennessee Health Science Center and were carried out in accordance with the principals of the Basel Declaration and standards of the Association for the Assessment and Accreditation of Laboratory Animal Care (AAALAC), as well as the recommendations of the National Institutes of Health Guide for the Care and Use of Laboratory Animals. The protocol was approved by the Institutional Animal Care and Use Committee (IACUC) at the University of Tennessee Health Science Center.

### Animals

All data used in this study came from mice that were part of the AD-BXD panel, which has been previously described (Neuner et al., 2018). Briefly, female B6 mice hemizygous for the 5XFAD transgene (B6.Cg-Tg(APPSweFlLon, PSEN1^∗^M146L^∗^L286V)6799Vas/Mmjax, Stock No. #34848-JAX) were mated to males from the BXD genetic reference panel (Peirce et al., 2004; Wang et al., 2016). As both of these resources (B6.5XFAD and individual BXD strains) consist of fully inbred mice, one generation of breeding results in isogenic F1 AD-BXD mice that harbor the 5XFAD transgene in combination with a genetically diverse BXD chromosome. As female 5XFAD mice are hemizygous, non-transgenic F1 mice were also generated (in approximately 50/50 ratio), but only results from 5XFAD positive F1 mice were included here, and mice are referred to as AD-BXD mice throughout the manuscript. Male and female AD-BXD mice were group housed with a mix of transgenic and non-transgenic same-sex littermates and maintained on a 12 h light/dark cycle.

### Contextual Fear Conditioning

Standard contextual fear conditioning (Neuner et al., 2015) was used to characterize cognitive function across the AD-BXDs at either 6 or 14 months of age. On the first day of training, mice were placed in a training chamber and four 0.9 mA 1 s foot shocks were delivered after a baseline period. Four post-shock intervals were defined as the 40 s following the offset of each foot shock and the percentage of time spent freezing during each interval was determined using FreezeFrame software (Colbourn Instruments, PA, United States). The percentage of time spent freezing following the final shock was used as a measure of contextual fear acquisition across the panel. Twenty-four hours after training, mice were placed back into the training chamber and the percentage of time spent freezing throughout the entire 10-min test was measured as an index of contextual fear memory. To evaluate the impact of genetic background, age, and sex on each of the contextual fear conditioning traits, a three-way ANOVA on individual-level data was run. Type III Sum of Squares for each term was compared to the SS value of the corrected total variance to calculate the percentage variance explained by each variable. For both contextual fear acquisition and contextual fear memory, strain background explained the majority of variance (19 and 15%, respectively – relative to 1% and 5% for age and 1% and 0.1% for sex). Strain/age/sex specific averages were generated to compare to RNA-sequencing and WGCNA results.

### RNA Sequencing

Initial RNA sequencing from hippocampus of the AD-BXD panel [5XFAD positive only; 6 months, *n* = 33 (15 females/18 males) and 14 months *n* = 36 (16 female/20 male)] has been previously reported [(Neuner et al., 2018), GEO accession number GSE101144]. Here, we expand upon this dataset and include RNA sequencing data from an additional 38 (32 female/6 male) 6 month and 50 (29 female/21 male) AD-BXD mice, for a total of 157 AD mice, 71 of which were 6 months of age (47 female/24 male) and 86 of which were 14 months of age (45 female/41 male). RNA-sequencing was performed as previously described (Neuner et al., 2018). Briefly, all samples were isolated using the Qiagen RNeasy Mini kit, libraries were prepared using Truseq Stranded mRNA Sample Preparation Kit (Illumina Inc.), and sequenced by 75 bp sequencing on an Illumina HiSeq2500. The GBRS pipeline was used to first align reads to a diploid B6/D2 transcriptome using Bowtie (Langmead et al., 2009) followed by an expectation maximization algorithm to quantify the number of reads aligned to either the *B* or *D* allele. The total number of reads assigned to a gene (across *B* and *D* alleles) was used here^1^. Genes were filtered to require an average of at least 1 transcript per million (TPM) in 50% of samples, RNA data was batch corrected using ComBat (Johnson et al., 2007; Leek et al., 2012), and biological replicates were averaged together for downstream analyses (Choi et al., 2017; Raghupathy et al., 2018). Specifically, samples from individual mice from the same strain, sex, and age were averaged together to derive one group average. Data from both GSE101144 and new data reported here (available now on GEO as GSE119215) represents data from 79 strain/sex/age groups across 28 background strains. Differential expression analysis comparing strain/sex averaged 6 month AD-BXD and 14 month AD-BXD gene expression was performed using DESeq2 (Love et al., 2014).

### Gene Set Enrichment Analysis

Gene set enrichment analysis (GSEA) was performed according to established procedures (Subramanian et al., 2005; Liberzon et al., 2011). Genes significantly differentially expressed relative to age in the AD-BXDs (14 months versus 6 months, adjusted *p*-value ≤ 0.05) were sorted by log_2_ fold change and uploaded to GSEA desktop software. Using the “GSEAPreranked” tool and the Molecular Signatures Database 3.0, Gene Ontology (GO) terms from all categories (Biological Process, Molecular Function, and Cellular Compartment) significantly enriched among either down-regulated or up-regulated genes were identified.

### Cell-Type Specific Enrichment Analysis

For cell-type specific enrichment analysis, a list of genes and their cell-type assignment based on max FPKM from single-cell RNA-sequencing in the mouse cerebral cortex was obtained from brainrnaseq.org (Zhang et al., 2014). A hypergeometric test was used to determine the statistical significance of overlap between our list of differentially expressed genes and lists of cell-specific genes. Only genes from differential expression that had a cell-type assignment were used; as such, the list of 12,978 genes downloaded from brainrnaseq.org was used as our background gene set for calculating statistical significance of overlap.

### Weighted Gene Co-expression Network Analysis

Weighted gene co-expression network analysis (WGCNA) was performed according to established methods (Langfelder and Horvath, 2008). Additional filtering required at least 1 TPM in 20% of samples, for a final gene list containing ∼16,000 genes. A minimum module size of 30 was implemented, and block-wise network construction was used to assemble modules using only 6 month-old AD-BXD RNA data. The WGCNA function GOenrichmentAnalysis was used to identify GO terms significantly enriched within each of the modules using a false discovery rate of 0.05. Hub gene identification using partial correlation analysis was performed using the Statistical Inference of Large-Scale Gaussian Graphical Model in Gene Networks (SILGGM) package in R (Zhang et al., 2018). Module eigengenes representing the first principal component of the expression matrix of the corresponding module were derived using standard methods within the WGCNA package (Langfelder and Horvath, 2008). These eigengenes were used as representative measures of gene expression profiles within a given module and are represented using arbitrary standardized units throughout the manuscript. To evaluate how increased module eigengene expression related to actual module member expression, we also derived a standardized module expression value from the mean expression level of all genes as previously reported (Mostafavi et al., 2018).

### Quantitative Trait Loci (QTL) Mapping

Genotypes for BXD strains were obtained from GeneNetwork.org (Mulligan et al., 2017). Module eigengenes as generated by WGCNA were exported and used as quantitative traits for downstream QTL mapping in r/qtl (Broman et al., 2003). Sex was used as an additive covariate and permutation tests were used to determine statistical significance.

### Statistical Analysis and Software

R software version 3.4.3 was used for data analysis. WGCNA version 1.63 was used for network analysis. Statistical tests included paired *t*-tests, ANOVA, hypergeometric tests for overlap, Spearman’s correlation, and permutation tests. Data are reported here as mean ± standard error unless otherwise stated.

## Results

### Inflammation and Loss of Synaptic Genes Underlie Population-Level Cognitive Decline in AD-BXDs

**FIGURE 1:**
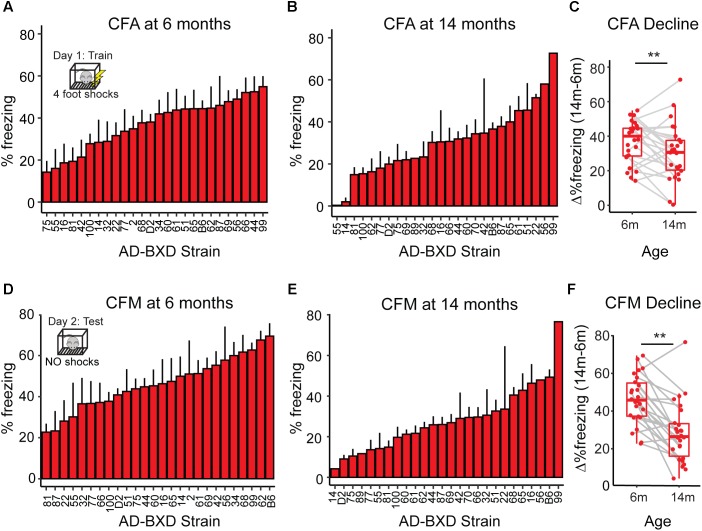
Cognitive function declines with age across genetically diverse AD mice. **(A)** At 6 months of age, heritable variation in contextual fear acquisition (CFA) exists across AD-BXDs, as measured by freezing following the final shock during the training trial of contextual fear conditioning. **(B)** Variation in CFA also exists at 14 months. **(C)** As a population, the AD-BXD panel declines significant in CFA performance with age [ANOVA main effect of age, *F*(1, 354) = 3.3, *p* < 0.001], although the extent of this decline varies widely by background strain. **(D)** At 6 months of age, heritable variation in contextual fear memory (CFM) exists across the AD-BXDs, as measured by total freezing during the 10-min testing trial of contextual fear conditioning. **(E)** Variation also exists at 14 months. **(F)** As a population, the AD-BXD panel declines significantly with age [ANOVA effect of age, *F*(1, 354) = 3.5, *p* < 0.001], although as with CFA, the extent of this decline varies widely across background strain. ^∗^Raw data originally reported in (Neuner et al., 2018). Each point in C and F represents a strain/age average.

**FIGURE 2:**
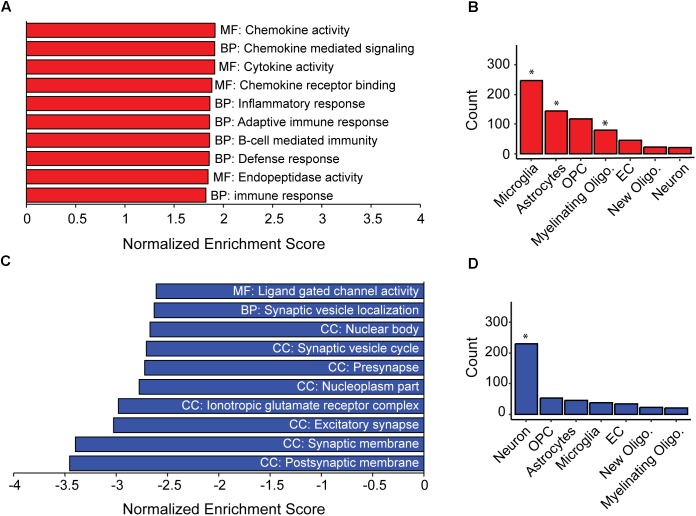
Inflammation and loss of synapses likely underlie population-level cognitive decline in AD-BXDs. **(A)** Gene set enrichment analysis (GSEA) identified a significant enrichment for gene ontology (GO) terms related to immune function among genes up-regulated with age across AD-BXDs. Designations before each term indicate category of GO term; MF, Molecular function, BP, Biological process, CC, Cellular compartment. **(B)** Genes upregulated in 14 month AD-BXDs that show cell-type specificity in their expression profiles showed enrichment for localization to microglia (*p* < 0.001), astrocytes (*p* = 0.02), and myelinating oligodendrocytes (*p* < 0.001), but not oligodendrocyte precursor cells (OPCs), endothelial cells (EC), newly formed oligodendrocytes (new oligo.), or neurons. **(C)** Among genes down-regulated in 14 month AD-BXDS, GSEA identified a significant enrichment for terms related to neuron function and in particular, synapse localization. **(D)** Genes upregulated in 14 month AD-BXDs that show cell-type specificity in their expression profiles showed enrichment for localization to neurons (*p* < 0.001). ^∗^*p* < 0.05.

As a population, the AD-BXDs significantly decline in cognitive function from 6 months to 14 months of age (Figure 1). In order to first understand the population-level transcriptional changes driving this decline, we profiled the transcriptome from a total of 157 AD-BXD mice, 71 of which were 6 months of age (47 female/24 male) and 86 of which were 14 months of age (45 female/41 male). Together, these mice represented a total of 79 sex/age groupings across 28 genetically diverse background strains, and biological replicates were averaged together for downstream analyses. Differential expression analysis using DESeq2 identified a total of 1278 genes that significantly change in expression (774 upregulated and 504 downregulated, adjusted *p*-value < 0.05) throughout the course of aging. Using gene set enrichment analysis (GSEA), a slight but significant upregulation of genes enriched for immune-related functions was observed (Figure 2A), suggesting neuroinflammation increases with age in the AD-BXDs. This increase seems to be driven by an increase both in microglia and astrocytes, as up-regulated genes that show cell-type specificity in their expression profiles showed enrichment for both microglia and astrocyte localization (Figure 2B, hypergeometric test, *p* < 0.0001 and *p* = 0.02, respectively). This up-regulation of immune-related genes mirrors changes observed in brain tissue collected post-mortem from human AD patients relative to controls (Zhang et al., 2013; International Genomics of Alzheimer’s Disease Consortium, 2015). Interestingly, there was also a significant enrichment among positively changed genes for expression in myelinating oligodendrocytes. In contrast, a robust downregulation of genes specifically enriched for localization to synapses, with functions involved in channel activity was observed (Figure 2C). As expected based on GO term enrichments, down-regulated genes that show cell-type specificity in their expression profiles showed enrichment for neuronal localization (Figure 2D, hypergeometric test, *p* < 0.0001), reminiscent of neurodegeneration observed in the classical 5XFAD model (Oakley et al., 2006) and human AD patients (Crews and Masliah, 2010).

### Characterization of Pre-symptomatic AD Transcriptional Network

**FIGURE 3:**
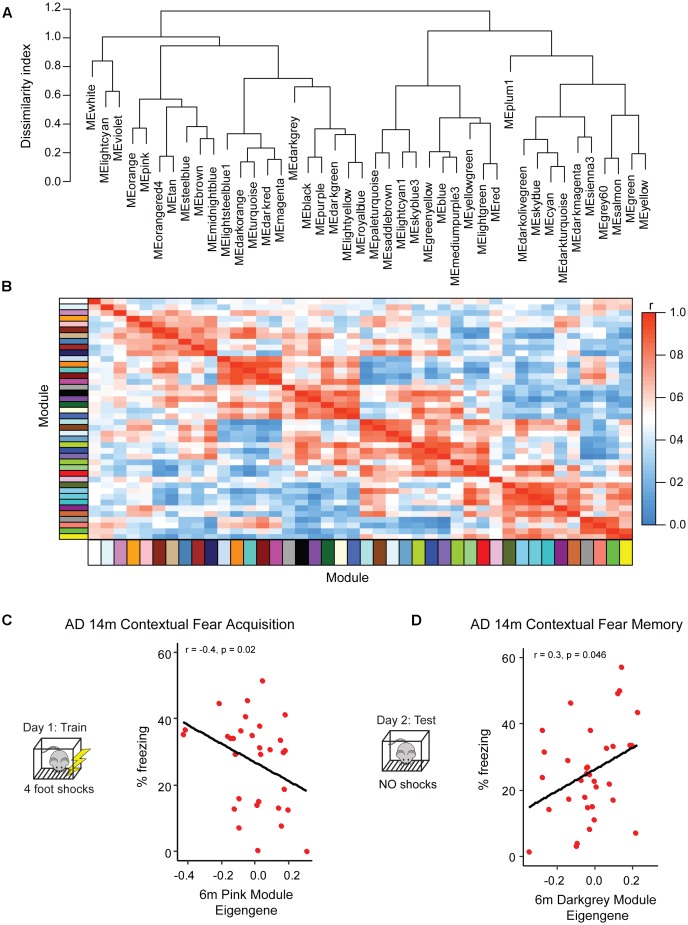
Identification of pre-symptomatic gene signatures predictive of AD resilience. To profile gene networks present in pre-symptomatic AD-BXDs, we performed weighted gene-co-expression network analysis (WGCNA) on hippocampal RNA-sequencing data. Modules were summarized by a single module eigengene (i.e., the first principal component) to simplify network visualization and analyses (Langfelder and Horvath, 2007). **(A)** Dendrogram depicting hierarchical clustering of all module eigengenes; dissimilarity indices were calculated by subtracting the r value for any given correlation from 1. **(B)** Heatmap illustrating correlation strength across the module eigengene network. **(C)** Expression of the 6 month pink module, as measured by the module eigengene, significantly correlates with strain-matched 14 month contextual fear acquisition (CFA). **(D)** Expression of the 6 month darkgray module significantly correlates with strain-matched 14 month contextual fear memory (CFM).

To begin to understand transcriptional networks present prior to overt symptom onset, we used weighted gene co-expression network analysis (WGCNA, Figure 3A,B) to identify modules of highly correlated genes present among genes that were expressed at a level of at least one transcript per million (TPM) in >20% of our samples. As groups of highly co-expressed genes presumably function in similar biological processes or pathways, module construction using only 6 month AD-BXD samples should provide a snapshot of the transcriptional profile within the pre-symptomatic population (Langfelder and Horvath, 2008). From WGCNA, we identified 43 modules ranging in size from 35 to 2038 genes. Of the 43 modules, 27 were enriched for at least one functional category (gene ontology, GO term) at FDR ≤ 0.05. A variety of GO terms were represented (Supplementary Table S1), including immune processes, neuronal function, mRNA binding, protein binding, and myelination, suggesting we are capturing a number of physiologically relevant processes occurring in young AD-BXD mice.

### Select Pre-symptomatic Gene Signatures Correlate With Cognitive Function Later in Disease

Although as a population the AD-BXDs decline with age, the extent of cognitive decline varies widely across the panel. Background strain explains a large portion of observed variation in cognitive decline (19% of total variance in contextual fear acquisition and 15% of the total variation in contextual fear memory) across 6 and 14 month-old AD-BXDs (Figure 1). To identify which, if any, of our identified modules correlate with this observed strain-specific variation in cognitive decline, we first summarized module expression using the module eigengene (ME) generated by WGCNA. In order to generate the ME, the expression of all genes in a given module is summarized via principal component analysis in order to obtain the first principal component, which explains the largest proportion of expression variation across a module. A ME is particularly useful as it reduces dimensionality of a module to a single representative measure, which then can be used to relate module expression to both genotypes and external phenotypes. Here, we correlate ME expression at 6 months to strain-matched cognitive performance at 14 months to obtain a quantitative measure of transcriptional networks that predict strain-specific susceptibility or resilience to AD-related cognitive decline. The module most highly correlated with contextual fear acquisition, as measured by freezing following the final shock on day 1, was the pink module (*r* = -0.4, nominal *p* = 0.02, Figure 3C), which was significantly enriched for over 200 GO terms at an FDR ≤ 0.05 (Supplementary Table S1). A vast majority of these terms involved immune system function, including immune system process, immune response, and regulation of cytokine production. Given the enrichment of immune-related terms among genes that increase with aging in the AD-BXDs (Figure 2A), the identification of an immune-enriched module as a critical predictor of late-life learning abilities suggests the ability of a strain to modify or fine-tune these processes as early as 6 months is critical for disease progression. A single 6 month module, the darkgray module (Figure 3D) was identified as highly correlated with 14 months contextual fear memory performance as measured by the percentage of time spent freezing throughout the 10-min test (*r* = 0.3, nominal *p* = 0.046). The darkgray module was also significantly enriched for a number of GO processes, although a smaller number than the pink module (*n* = 50). A large number of these GO terms related to extracellular matrix organization, extracellular organelle and vesicle localization, and aspects of protein binding (integrin binding, heparin binding, calcium ion binding, etc.). Interestingly, the identification of the darkgray module also highlights a putative role for brain vasculature in disease progression, as the module was significantly enriched in the GO terms blood vessel development, circulatory system development, cardiovascular system development, blood vessel morphogenesis, and angiogenesis (*FDR* ≤ 0.05).

### Identification of Specific Drivers of Module-Trait Associations

**FIGURE 4:**
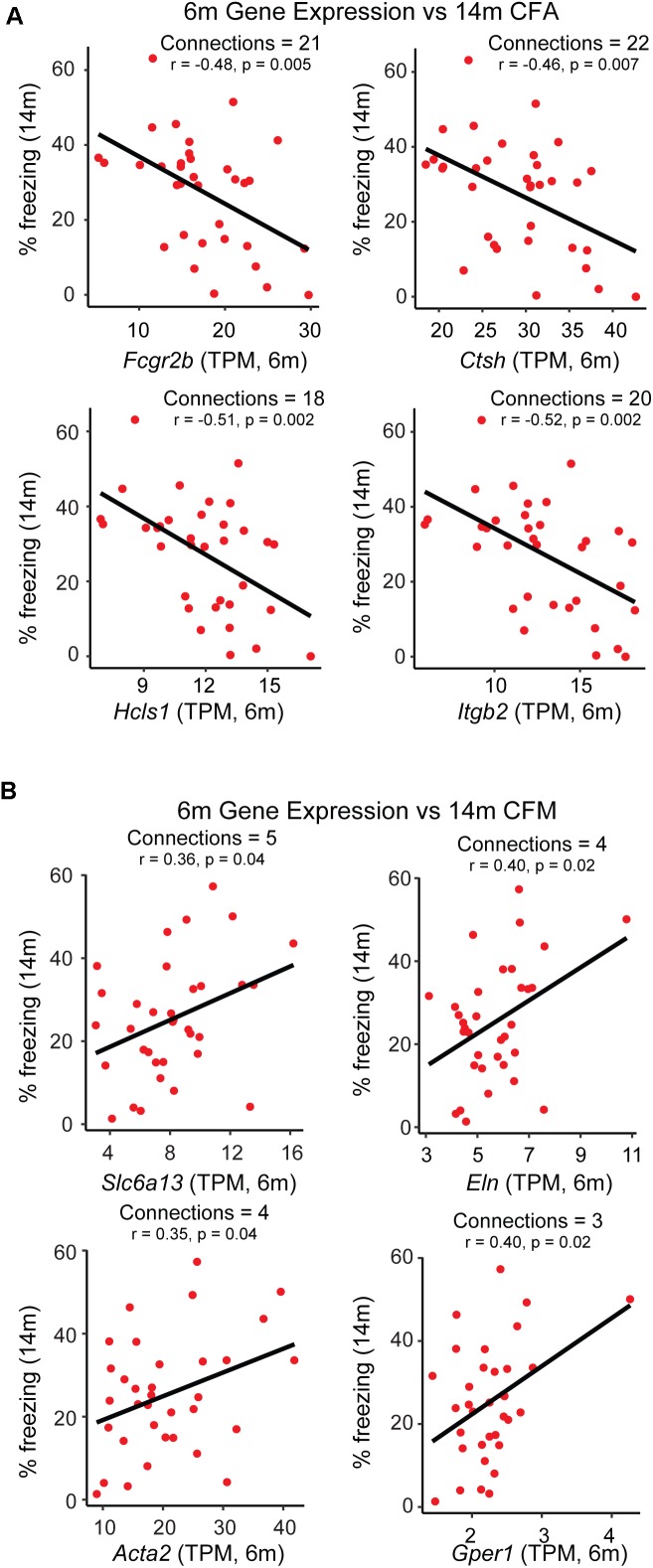
Identification of putative candidate genes that may be targeted to promote resilience networks. A combination of hub gene identification via partial correlation analysis and single-gene correlation prioritization was used to identify likely candidates driving the association between 6 month modules and 14 month cognitive performance. **(A)** Gene expression of putative candidates *Fcgr2b*, *Ctsh*, *Hcls1*, and *Itgb2* at 6 months is plotted against strain-matched 14 month contextual fear acquisition (CFA). **(B)** Gene expression of putative candidates *Slc6a13*, *Eln*, *Acta2*, and *Gper1* at 6 months is plotted against strain-matched 14 month contextual fear acquisition (CFM). TPM = transcripts per million.

While GO enrichment analyses give a broad snapshot of specific functions and pathways that may be targeted to promote resilience, one of the strengths of network analysis is that specific ‘hub genes’, or drivers of intra-modular connectivity, can be identified. These genes represent ideal therapeutic targets with which to manipulate the transcriptome on a modular level, as they are highly connected to other genes within a given module. However, traditional hub gene identification in WGCNA relies simply on a measure of gene-gene correlation (Langfelder and Horvath, 2008), and sometimes identifies a number of indirect connections such that if gene A regulates gene B and gene B regulates gene C, gene A and C will be appear to be highly connected based on their mutual relationship with gene B. To limit the use of indirect connections in nominating hub genes, we utilized partial correlation analysis (Zhang et al., 2018), where measures of gene-gene correlations are obtained by conditioning on all other genes in the module, such that the only genes that would appear to be connected in the previous scenario are genes A and B and genes B and C, but not genes A and C. It was our hypothesis that this approach would nominate hub gene candidates that could be targeted to most efficiently and directly modulate the broader network. In addition to hub genes, we were interested in module members highly correlated with the specific trait of interest themselves, independent of relationship to the ME or other module members. In order to come up with a list of candidates that may be targeted to promote networks hypothesized to be underlying resilience (i.e., the pink and darkgray modules), we selected genes appearing both in the list of top 30 hub genes remaining after partial correlation analysis and which exhibited significant correlation with either contextual fear acquisition or memory or at a *p*-value ≤ 0.05. The top 4 candidates from each module were identified and their relationship with 14 m cognitive performance is highlighted in Figure 4. A number of candidates with known links to AD were identified, including low affinity immunoglobulin gamma Fc region receptor II-b (*Fcgr2b*) (Kam et al., 2013), as well as candidates not well studied in the context of aging or AD, including G-protein coupled estrogen receptor 1 (*Gper1*) (Briz et al., 2015), both validating our approach and identifying novel candidates that likely contribute to disease onset.

### Genomic Region on Chromosome Two Underlies Variation in Resilience Networks

**FIGURE 5:**
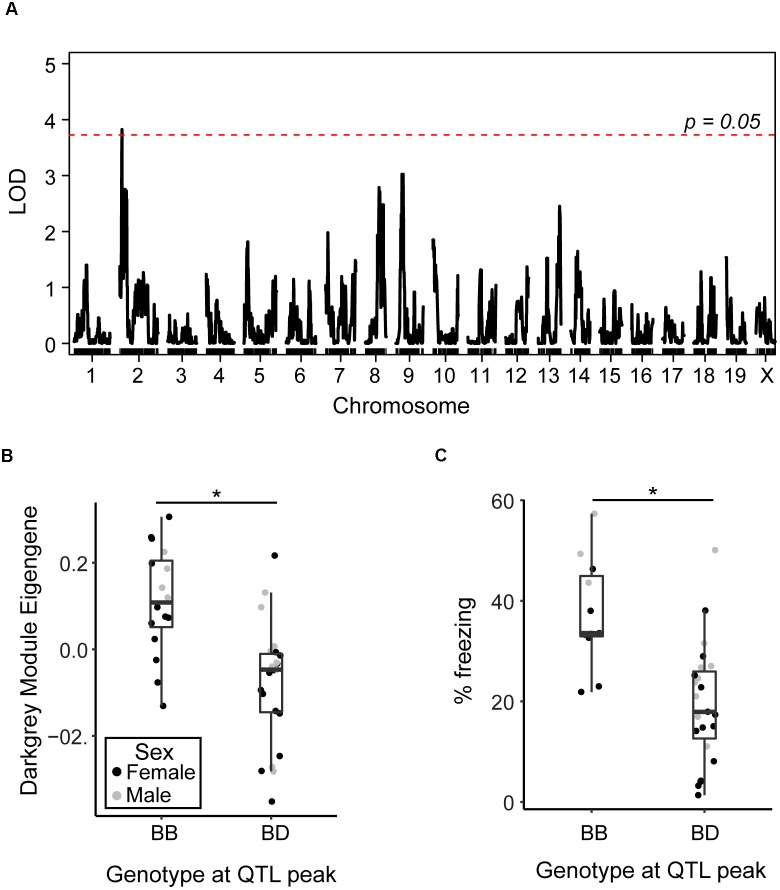
Identification of genomic regions underlying variation in resilience networks. **(A)** A significant quantitative trait locus (QTL) was identified as regulating the expression of the darkgray module (LOD = 3.8, 1.5 LOD interval = 15–44.5 Mb). **(B)** Strains carrying the *D* allele at the peak QTL marker exhibited significantly lower expression of the darkgray module [*t*(1, 37) = 4.4, *p* < 0.001]. **(C)** Strains carrying the *D* allele at the peak QTL marker with strain-matched 14 month cognitive data exhibited significantly worse contextual fear memory [CFM, *t*(1, 32) = 4.3, *p* = 0.0002]. ^∗^*p* < 0.05.

As the AD-BXD population was derived from the BXD genetic reference panel, which has been densely genotyped, we can begin to investigate the contribution of specific genetic variants to observed phenotypes, including the variation in transcriptional networks described here. In order to identify regions of the genome involved in regulating observed variation in identified resilience networks, we performed quantitative trait loci (QTL) mapping using either the pink or darkgray ME as a quantitative trait. No QTLs were identified for the pink module, but there was a significant QTL on chromosome 2 identified as a regulator of the darkgray module (Figure 5A). Strains carrying a copy of the D2 (*D*) allele at the peak QTL marker had lower expression of the darkgray module, as represented by lower ME values (Figure 5B), and lower contextual fear memory performance at 14 m (Figure 5C). We confirmed higher ME values were associated with higher module member expression by relating the mean standardized expression level of all member genes (Mostafavi et al., 2018) to the WGCNA-derived eigengene (*r* = 0.93, *p* < 0.01). These results demonstrate the presence of genetic variants in the region that significantly modify the expression of the group of genes that make up the darkgray module and contribute to variation in cognitive decline. The identified interval is large (∼30 Mb), so pinpointing the causal variant is difficult. Three module members, sperm associated antigen 6 (*Spag6*), olfactomedin-like 2A (*Olfml2a*), and prostaglandin D2 synthase (*Ptgds*) appear in the QTL interval, although as they were not top hub genes identified via partial correlation analysis, it’s unclear if they play a causal role in regulating module expression. These positional candidates are highly correlated with hub genes *Slc6a13* and *Eln*, suggesting they associate more strongly with the darkgray CFM module than the pink CFA module. Exactly, how genes and variants in the QTL regulate module expression remain to be elucidated; any number of positional candidates may play a role in transcription and gene regulation at the protein level rather than transcriptional level. Alternatively, a number of micro-RNAs (miRNAs) and long non-coding RNAs (lncRNAs) exist in the identified interval and may post-transcriptionally regulate module gene expression.

## Discussion

### Utility of the AD-BXDs for Studying Resilience

Resilience to AD, defined as better than expected cognitive functioning based on high pathology load or high-risk genetic background, has traditionally been difficult to study, both in mice and humans. Specifically, human individuals with intact cognitive functioning rarely enter the clinic, and if they do, lack of access to relevant brain tissue early in disease precludes the identification of causal molecular mediators of cognitive decline. Studies in the mouse have traditionally utilized only a single AD model (Onos et al., 2016), where genetic variance and phenotypic variance is low relative to a highly penetrant AD transgene. We overcome some of these barriers by utilizing the genetically diverse AD-BXD mouse population, as this series of genetically diverse inbred mice show highly variable susceptibility to disease (Neuner et al., 2018). All of the AD-BXD mice harbor a high-risk genotype in the form of the 5XFAD mutation, however not all strains show the expected degree of cognitive decline that is typically seen as a result of the aggressive 5XFAD transgene (Oakley et al., 2006). Instead, a number of strains demonstrate a certain degree of cognitive resilience (Figure 1). Given the inbred nature of the panel, the AD-BXDs represent an ideal opportunity to study the molecular determinants of resilience, as we now have the opportunity to obtain brain tissue early in disease while repeatedly sampling genetically identical individuals later in life in order to phenotypically classify certain strains as either susceptible or resilient to disease onset and better understand which individuals would have gone on to develop severe AD dementia.

### Hub Genes as Putative Candidates for Promoting Resilience

Here we identify two modules, the pink module and the darkgray module, as gene networks present early in disease that correlate with cognitive function later in life. It is our hypothesis that the biological pathways and processes represented by these modules play a role in priming the brain for late-life susceptibility or resilience to AD. As such, we hypothesize that manipulating the expression of either of these modules in the desired direction (i.e., down-regulating the pink module or up-regulating the darkgray module) early in life would maintain cognitive function late in disease, as observed in our resilient strains. Hub genes, or highly connected gene within a given module, represent viable targets to manipulate whole-module expression, and they likely influence expression of a number of their nearest neighbors. Here we nominate the top four candidates from each module as putative candidates that may be targeted to promote cognitive resilience. As the pink module was largely enriched for immune-related GO terms, a number of the candidates that emerged from this module related to immune function. Although neuroinflammation has repeatedly been identified as a point of therapeutic intervention in AD (Bronzuoli et al., 2016), our approach now provides precise targets that may be leveraged early in disease. One target, integrin subunit beta 2 (*Itgb2*) has been identified as a member of the module identified by Zhang et al. (2013) to be most highly associated with AD onset in human patients, suggesting that target in particular harbors translational relevance to human disease. Interestingly, the candidates nominated by prioritization of members in the darkgray module have more varied roles, as suggested by the more diverse GO terms enriched within the darkgray module. Two candidates, elastin (*Eln*) and actin, alpha 2, smooth muscle, aorta (*Acta2*) again highlight the critical role of brain vasculature in the maintenance of cognitive function, as *Eln* is a major structural component of arterial walls and *Acta2* has been implicated in vascularization and vascular branching (Rudnicki et al., 1990; Yamada et al., 1999; Wagenseil and Mecham, 2012). The two remaining candidates, solute carrier family 6 member 13 (*Slc6a13*) and *Gper1*, are both ion channels and receptors that have been implicated in neuronal signaling and synaptic plasticity (Briz et al., 2015; Chazalon et al., 2018). Of particular interest from the darkgray module is the gene *Slc6a13*, as this gene, a GABA transporter, has been identified as a potential blood biomarker in AD (Long et al., 2016), suggesting this gene (in addition to *Itgb2*) may have translational relevance to human disease. In summary, results here show that dampening specific immune system processes while enhancing vascularization and synaptic health early in life may promote cognitive resilience later in disease. In combination with specific candidate gene prioritization, this analysis now provides specific candidates on which to focus future studies.

### Identification of Genetic Mediators of Resilience

Here we show that genetic background explains a large portion of variation (∼15–20%) in cognitive functioning among AD-BXD strains. We then go on to identify a specific region on chromosome 2 that directly influences expression of one of our identified resilience modules, the darkgray module. While the region was too large in the mouse to narrow down specific gene variants involved in modifying resilience, a bulk of evidence suggests this region is relevant to AD in human populations as well. Portions of the identified QTL are syntenic to a region of chromosome 9 (specifically 9q34) in the human genome, and evidence that chromosome 9 may harbor variants that influence AD susceptibility has emerged from a number of linkage studies in FAD populations. Specifically, 9q34 was identified as linked to AD onset in autopsy-confirmed familial AD patients in 1999 (Kehoe et al., 1999) and again in a study using 466 FAD families in 2000 (Pericak-Vance et al., 2000). Future studies that add additional AD-BXD strains in order to increase mapping power may be able to narrow this genomic interval and identify causal gene variants, which would better inform human studies seeking to identify novel AD genes. As none of our identified hub genes reside in this QTL, it is likely that the genes located within the QTL exert their effect on module expression at the protein level, a possibility that will be examined in future studies. In addition, a number of non-protein coding genes exist in the interval, such as micro-RNAs and long non-coding RNAs. These types of molecules have been shown to exert large effects on transcriptional networks (Gamazon et al., 2012; Jalali et al., 2013; Brown et al., 2014), but were largely excluded from the WGCNA module assembly, as our RNA isolation and library preparation protocol targeted poly-A enriched mRNAs. Future work will examine the possibility that genetic variation in a number of these non-coding genes may work to influence transcriptional networks underlying AD resilience.

### Conclusions and Future Directions

Overall, work presented here takes advantage of the significant opportunity provided by the AD-BXDs and the reproducibility inherent to utilizing inbred mouse strains in a well-controlled environment to make one of the first attempts to map the transcriptional network underlying cognitive resilience to AD. Our results suggest cognitive resilience results from fine-tuned balance between low inflammation levels and high vascular and synaptic function early in life, and provides not only general pathways but specific candidates that may represent valuable intervention points in future studies. Numerous validation studies will need to be carried out to confirm or refute the hypotheses presented here, but these studies will be greatly facilitated due to the rigor and reproducibility afforded by the AD-BXD panel. As there is currently no cure for AD, understanding the mechanisms that protect some individuals from developing this devastating disease would be instrumental in developing novel therapeutics and ultimately, finding a cure.

## Data Availability Statement

The RNA-seq generated here is available on Gene Expression Omnibus (GEO) as GSE119215. RNA data previously reported (Neuner et al., 2018) is available on GEO as GSE101144. All raw data, including raw phenotype information, has been deposited in the AMP-AD Knowledge Portal synapse at the following link: https://doi.org/10.7303/syn17016211. Strain-averaged behavioral data has been deposited on GeneNetwork.org and is available as Record IDs 20473-20964.

## Author Contributions

SN and CK conceived of the experiments, designed the experiments, and wrote the manuscript. J-GZ and SN conducted bioinformatics experiments. SN, SH, VP, and CK assisted in data analysis and interpretation. All authors read and approved of the final manuscript.

## Conflict of Interest Statement

The authors declare that the research was conducted in the absence of any commercial or financial relationships that could be construed as a potential conflict of interest.

## Footnotes

1 https://github.com/churchill-lab/gbrs
